# Effective Osteogenic Priming of Mesenchymal Stem Cells through LNA-ASOs-Mediated *Sfrp1* Gene Silencing

**DOI:** 10.3390/pharmaceutics13081277

**Published:** 2021-08-17

**Authors:** Daniel García-Sánchez, Alberto González-González, Patricia García-García, Ricardo Reyes, María Isabel Pérez-Núñez, José A. Riancho, Carmen Évora, José Carlos Rodríguez-Rey, Flor M. Pérez-Campo

**Affiliations:** 1 Department of Biochemistry and Molecular Biology, Faculty of Medicine, University of Cantabria-IDIVAL, 39012 Santander, Spain; daniel.garciasa@alumnos.unican.es (D.G.-S.); alberto.gonzalezgo@alumnos.unican.es (A.G.-G.); josecarlos.rodriguez@unican.es (J.C.R.-R.); 2 Department of Chemical Engineering and Pharmaceutical Technology, Institute of Biomedical Technologies (ITB), University of La Laguna, 38206 La Laguna, Spain; patg0991@gmail.com (P.G.-G.); cevora@ull.es (C.É.); 3 Department of Biochemistry, Microbiology, Cellular Biology and Genetics, Institute of Biomedical Technologies (ITB), University of La Laguna, 38200 La Laguna, Spain; rreyesro@ull.edu.es; 4 Department of Traumatology, Hospital Universitario Marqués de Valdecilla, University of Cantabria, 39008 Santander, Spain; isabel.perez@unican.es; 5 Department of Internal Medicine, Hospital Universitario Marqués de Valdecilla-IDIVAL, University of Cantabria, 39012 Santander, Spain; jose.riancho@unican.es

**Keywords:** mesenchymal stem cells, bone regeneration, LNA-ASO, GapmeR, *Sfrp1*, osteogenesis, BMP

## Abstract

Mesenchymal stem cell (MSC) transplantation has emerged as a promising approach for bone regeneration. Importantly, the beneficial effects of MSCs can be improved by modulating the expression levels of specific genes to stimulate MSC osteogenic differentiation. We have previously shown that *Smurf1* silencing by using Locked Nucleic Acid-Antisense Oligonucleotides, in combination with a scaffold that sustainably releases low doses of BMP-2, was able to increase the osteogenic potential of MSCs in the presence of BMP-2 doses significantly smaller than those currently used in the clinic. This would potentially allow an important reduction in this protein in MSs-based treatments, and thus of the side effects linked to its administration. We have further improved this system by specifically targeting the Wnt pathway modulator *Sfrp1*. This approach not only increases MSC bone regeneration efficiency, but is also able to induce osteogenic differentiation in osteoporotic human MSCs, bypassing the need for BMP-2 induction, underscoring the regenerative potential of this system. Achieving successful osteogenesis with the sole use of LNA-ASOs, without the need of administering pro-osteogenic factors such as BMP-2, would not only reduce the cost of treatments, but would also open the possibility of targeting these LNA-ASOs specifically to MSCs in the bone marrow, allowing us to treat systemic bone loss such as that associated with osteoporosis.

## 1. Introduction

Mesenchymal stem cells (MSCs) are multipotent cells present in different adult tissues that possess the capacity to differentiate into various specialized cell lineages including osteoblasts, adipocytes, and chondrocytes. Due to this ability, in the last few years, there has been an increasing interest in using MSCs-based approaches to improve bone repair and regeneration [1]. In particular, the use of MSCs-based therapies would benefit the treatment of critical size bone defects resulting from direct trauma or from the removal of large bone areas through surgical procedures in patients with osteosarcoma, osteonecrosis, or other pathologies. Due to the known drawbacks of autologous and allogeneic bone graft, bone tissue engineering has emerged as an interesting alternative. One of the main obstacles for the development of a successful MSCs-based therapy is to obtain an efficient osteogenic differentiation of the transplanted cells. Different MSCs modifications have been designed to achieve this point [2,3,4,5].

Due to their key function in bone formation, the BMP and Wnt/β-catenin signaling pathways are interesting targets for the development of therapies aimed to treat bone loss associated diseases. We have previously shown that the sustained activation of the BMP pathway through the silencing of *Smurf1* (SMAD specific E3 Ubiquitin Protein Ligase 1), a major inhibitor of this pathway, has a marked positive effect on bone formation [5,6]. Importantly, this was achieved by using a particular class of Locked-Nucleic-Acids Antisense-Oligonucleotides (LNA-ASOs) known as GapmeRs, a clinically safe method that would enable the translation of this protocol to the clinical practice. Moreover, using this approach, we were able to overcome the reduced osteogenic potential showed by MSCs from osteoporotic patients [7]. These results were achieved in the presence of BMP-2 concentrations one million times lower than those used in the clinic [6], opening the possibility of significantly reducing the adverse effects linked to high doses of these factors [8,9,10,11,12,13,14], one of the problems hindering the clinical use of regenerative techniques in this field. Whereas the osteogenic induction using the previous method was highly significant, the overall increase in bone formation in vivo through the silencing of *Smurf1* in MSCs did not exceed 30%. In this current work, we set out to increase this efficiency by targeting other extra and intracellular osteogenic inhibitors.

We hypothesize that it would be possible to achieve a greater pro-osteogenic effect by silencing genes coding for other negative regulators of this and other osteogenic pathways, either independently or in combination with *Smurf1*. *Tob1* (Transducer of Erb-2 1) is one of the genes that could be useful for this purpose. This gene encodes an inhibitor of the interaction of RUNX2 and SMADs complexes, switching off the osteogenic signal initiated by the binding of BMP proteins to their receptors. Importantly, *Tob1* defective mice show abnormally high levels of bone formation and block osteoporosis induced by estrogen deficiency [15]. Other signaling networks are coordinated with BMPs to regulate the osteogenic differentiation of MSCs. The one receiving more attention is the Wnt/β-catenin pathway. In fact, several molecules involved in the regulation of Wnt signaling, such as sclerostin, are currently being targeted in bone-building therapies for patients with osteoporosis [16]. The key switch of this pathway is β-catenin, which, upon binding of Wnt to Wnt receptors, translocates into the nucleus and forms a dimer with LEF (Lymphoid Enhancer Binding Factor). This dimer acts as a transcription factor to promote *Runx2* transcription and the differentiation of MSCs to osteoblasts [17]. Data from several rare mutations affecting bone mass and from knockout mice [18] have revealed two more genes whose silencing would enhance bone formation. One encodes for the secreted frizzled related protein 1 (*Sfrp1*), that antagonizes canonical Wnt signaling by binding to Wnt ligands, preventing their binding to the receptors and inhibiting their downstream signaling activity [19]. Mice defective in *Sprf1* have a higher bone mass and *Sfrp1*^-/-^ mouse progenitor cells are directly shifted into the osteoblastic lineage. Interestingly, *Sfrp1* has been already proposed as a drug target for the treatment of fracture healing [20]. On the other hand, bone of osteoporotic patients shows an enhanced differentiation of MSCs to adipocytes [21]. The product of the gene Chibby Family Member 1 (*Cby1*), a nuclear protein that acts as an intracellular antagonist of the Wnt canonical pathway, prevents the formation of the β-catenin/Lef/Tcf [22] complex, directing multipotent progenitor cells toward the adipocytic differentiation. Since the osteogenic and adipogenic fates of MSCs are mutually exclusive, *Cby1* silencing could block adipogenic differentiation directing MSCs toward osteoblast formation.

In this current work, we tested whether the silencing of genes encoding for inhibitors of the BMP signaling pathway others than *Smurf1* (*Tob1*) and inhibitors of the Wnt/β-catenin pathway (*Cby1* and *Sfrp1*) in MSCs could, either individually or in combination, potentiate the osteogenic priming of MSCs and promote bone mass growth in a more efficient way than the sole silencing of *Smurf1*. However, since the Wnt/β-catenin pathway is crucial not only for determining cell fate, but also in the regulation of several cell cycle events, we first needed to verify that the silencing of Wnt/β-catenin inhibitors did not alter key processes such as cell proliferation, cell death or cell migration. Additionally, since there is increasing evidence that the canonical and non-canonical Wnt pathways are intersecting networks, we also needed to verify that silencing of the inhibitors of the Wnt/β-catenin pathway selected for this work (*Cby1* and *Sfrp1*) did not influence the activity of the non-canonical Wnt pathways.

We describe here how *Sfrp1* silencing in MSCs can considerably accelerate bone formation both in vitro and in vivo in an ectopic mouse model, something that could be advantageous in clinical practice, for example, to reduce the length of fracture healing. Importantly, the pro-osteogenic effect obtained by the silencing of *Sfrp1* significantly exceeds that achieved by *Smurf1* silencing, particularly in an in vivo setting [6]. Although alteration of the expression levels of *Sfrp1* in some cell lineages has been linked to different pathological conditions including tumor formation, we show here that transiently silencing *Sfrp1* in MSCs does not seem to lead to any abnormal biological behavior that could compromise the biosafety of these cells. Additionally, no concomitant activation of other Wnt pathways has been detected. Besides its greater osteogenic efficiency, the use of *Sfrp1* silencing for enhancing bone formation has an additional advantage over the silencing of *Smurf1*, since *Sfrp1* silencing using LNA-ASOs in MSCs is able to achieve a similar degree of osteogenic induction to that attained by direct BMP-2 stimulation. As BMP-2 is not needed to ensure the activation of the Wnt/β-catenin pathway, this approach would completely avoid the potential side effects currently associated with the use of this recombinant protein in the clinic [23] and considerably reduce the cost of treatments. Our results also highlight the efficacy of the inhibitors of the Wnt/β-catenin pathway in bone regeneration.

## 2. Materials and Methods

### 2.1. Cell Isolation

Human MSCs were isolated from femoral heads of osteoporotic patients undergoing hip replacement surgery due to an osteoporotic fracture, following previously described protocols [7]. A reduced number of colonies (on average < 10) appeared between five and seven days after seeding. Once the initial culture was 80–90% confluent, cells were expanded to achieve a final cell number between 3–5 × 10^5^. Due to the risk of those cells undergoing replicative senescence with subsequent passages, only cells in passage 1 were routinely used for all experiments.

A total of five samples obtained from female patients aged 65 to 85 were included in the study. The study protocol (Identification Code 2016.159) was approved on 17 October 2016 by the Institutional Review Board (Comité Ético de Investigación Clínica de Cantabria).

### 2.2. Cell Culture and Differentiation

The immortalized murine MSC line C3H10T1/2 was cultured in Dulbecco’s Modified Eagle’s Medium (DMEM, Invitrogen, Waltham, MA, USA) supplemented with 10% FBS and 1% penicillin-streptomycin. For osteogenic differentiation, C3H10T1/2 cells were plated at 12,500 cells/cm^2^ and hMSCs at 20,000 cells/cm^2^, allowing them to attach overnight. To induce differentiation, culture medium was replaced with osteogenic media 24 h later. C3H10T1/2 were differentiated with DMEM supplemented with 50 µM ascorbic acid, 20 Mm β-glycerolphosphate, and 1 µM dexamethasone. Human primary MSC osteogenesis was induced with DMEM supplemented with 50 µM ascorbic acid, 10 Mm β-glycerolphosphate, and 0.1 µM dexamethasone.

For C3H10T1/2 adipogenic differentiation, culture medium was replaced with adipogenic media. DMEM (10% FBS and 1% penicillin-streptomycin) supplemented with 1 µM dexamethasone, 2 µM rosiglitazone, 5 µg/mL insulin, and 0.5 mM isobutyl methylxanthine (IBMX).

### 2.3. GapmeRs Design

Antisense LNA GapmeRs were purchased from Exiqon (Qiagen, Venlo, The Netherlands). As a control, an Antisense LNA GapmeR Negative Control A (Ref. 339516) was used.

### 2.4. Flow Cytometry

A FACSCanto II flow cytometer and FACSDiva Software version 6.1.2 (BD Bioscience, San Diego, CA, USA) were used for the cytometry assays. GapmeR delivery efficiency after lipofection was tested as described in [6] by using FAM-Control labeled GapmeRs.

For the apoptosis assay, FITC Annexin V Apoptosis Detection Kit I (556547, BD Bioscience, San Diego, CA, USA) was used following the protocol described by the manufacturer.

### 2.5. Cell Transfection

Lipofection was performed using Dhamafect (Dharmacon, Horizon Discovery, Cambridge, UK). following instructions from the manufacturer. The C3H10T1/2 cell line was seeded at 12,500 cells/cm^2^, and human primary cells at 20,000 cells/cm^2^. Two hours prior to transfection, culture media was replaced with Opti-MEM I media (Invitrogen, Waltham, MA, USA). In order to increase transfection efficiency, no serum supplementation was added to these media for the transfection procedure. The recommended amount of Dharmafect was mixed with Opti-MEM I. On the other hand, to prepare the GapmeR mix, each of the GapmeR or GapmeRs combinations was also mixed with Opti-MEM I. After 5 min of incubation at room temperature (RT), GapmeR mix was added dropwise on top of the Dharmafect mix, and incubated for 20 min at RT. The mix was then added to the cells dropwise. Twenty-four hours after transfection, one volume of DMEM with 10% FBS and 1% penicillin-streptomycin was added to the wells and incubated at 37 °C for another 24 h. Forty-eight hours after transfection, cells were washed once with PBS and fresh complete culture media was added.

For in vivo experiments, right after transfection, 50,000 cells were seeded onto discoidal alginate scaffolds prepared as previously described [6]. Cell-loaded scaffolds were incubated a minimum of 20 min to allow cell attachment to the scaffold prior to implantation in the recipient mice. Longer incubation times (up to 24 h) could also be applied if necessary. In this occasion, cells were incubated with the scaffold for 14 h prior implantation.

### 2.6. Proliferation Assay

For the proliferation assay, cells were transfected in 6-well plates and harvested 48 h after transfection. After estimation of cell number, different cell concentrations (100, 200, 300, 400 cells per well) were seeded onto a 96-well plate in triplicate and allowed to attach overnight. Then, culture medium was changed, and cells were allowed to proliferate for the required number of days. Plates were harvested at days 1, 3, 5, 7, and 9. To determine cell viability, the medium was changed to DMEM (10% FBS and 1% penicillin-streptomycin) containing 0.5 mg/mL of 3-(4,5-dimethylthiazol-2-yl)-2,5-diphenyltetrazolium bromide (MTT). Cells were incubated 4 h at 37 °C. After this incubation, the medium was removed, 100 μL of 2-propanol was added to the wells, and the plate was incubated at 37 °C for 10 min. When the incubation time was finished, absorbance at 570 nm was measured with a plate lector Eon (BioTek, Winooski, VT, USA).

For the Ki67 immunofluorescence assay, cells were seeded on 10 mm diameter crystals, pretreated with gelatin 0.1%, then transfected as described above. A Ki-67 monoclonal antibody (SP6: MA5-14520, Invitrogen, Waltham, MA, USA) was used as a primary antibody. A mouse anti-rabbit secondary antibody labeled with FITC was used for subsequent detection (sc-2359, Santa Cruz Biotechnology Inc., Dallas, TX, USA). Crystals were observed under a fluorescence microscope, where pictures were taken and analyzed with ImageJ 1.53 c software (http://imagej.nih.gov/ij) to score positive cells.

### 2.7. Migration Assay

To study cell migration capability, a wound healing assay was performed. For this, cells were seeded at a high confluence in 6-well plates (25,000 cells/cm^2^), and transfected following the protocol already described. Forty-eight hours after transfection, MesenPro medium was added to the cells and 400 µM wide wounds were performed in each plate. Pictures were taken at 3 h intervals for 72 h. For each of the samples, six different areas along the wound were recorded and analyzed. Wound area was measured every 12 h. Images were analyzed using the ImageJ 1.53 software to evaluate the wound size by quantification of the empty areas occupied by the lesion.

### 2.8. Staining Quantification

Alizarin Red quantification was performed following a previously described method [24]. Oil Red quantification was also performed following previously described protocols [25]. Absorbance lecture of the plates was performed using an Eon Microplate Spectrophotometer (BioTek, Winooski, VT, USA).

### 2.9. Gene Expression

mRNA was isolated from cell cultures, after washing cells twice with PBS and collecting them with TRIzol reagent (Invitrogen, Waltham, MA, USA) by scraping the plate surface. RNA extraction was done following the protocol of the TRIzol manufacturer. Between 1 and 1.5 μg of RNA were isolated from a confluent 24-well plate. RNA retro-transcription was performed with the PrimeScript RT Reagent Kit (RR037A, Takara Bio Inc, Shiga, Japan) following the manufacturers’ instructions. A total of 500 ng of RNA was used in a 10 μL reverse transcription reaction. The resulting cDNA was diluted four times with ddH2O. One μL of this solution was used for each PCR reaction.

To measure the gene expression levels, semi-quantitative PCRs were performed using Taqman assays (Applied Biosystems, Waltham, MA, USA). To test gene silencing in the murine cell line C3H10T1/2, the following Taqman assays were used: Mm00503802_m1 (*Cby1*), Mm00489161_m1 (*Sfrp1*), Mm00547102_m1 (*Smurf1*), and Mm01496371_m1 (*Tob1).* To analyze gene expression of the *Runx2*, *Alpl*, *Bglap*, *Lpl*, and *Pparg* genes, the following assays were used: Mm00501578_m1, Mm01187117_m1, Mm03413726_mH, Mm00440940_m1, and Mm00434764_m1, respectively. Mouse housekeeping genes *GAPDH* (Assay Mm99999915_G1) and *RPL13A* (Assay Mm0162986_gH) were used for normalization.

For the gene expression analyses in the primary hMSCs the following Taqman assays were used: Hs00360360_m1 (*Cby1*), s00610060_m1 (*Sfrp1*), Hs00410929_m1 (*Smurf1*), Hs03986111_s1 (*Tob1*), Hs00231692_m1 (*Runx2*), Hs00758162_m1 (*Alpl*), and Hs01587814_g1 (*Bglap*). Human *GAPDH* (Hs99999905_m1) and *RPL13A* (Hs04194366_g1) genes were used for normalization.

Amplification conditions for all experiments were as follows: One initial denaturation cycle of 10′ at 90 °C, followed by 40 cycles with 15 seconds denaturation at 90 °C and 1 min extension at 60 °C.

### 2.10. Western Blot Analysis

To prepare the protein extracts, 1.2 million cells per condition were transfected. Forty-eight hours after transfection, cells were washed, harvested, and centrifuged. The supernatant was discarded, and cells were resuspended in 100 µL per million of cells of Pierce RIPA buffer (89900, Pierce Biotecnology, Waltham, MA, USA) supplemented with protease inhibitors to obtain a cell extract. On average, 100 μg of proteins were obtained for each of the conditions. Routinely, 15 to 20 μg of protein extract per well were loaded onto a precast NuPAGE 4–12% Bis-Tris Gel (Invitrogen, Waltham, MA, USA).

For the cytosolic and nuclear protein fractions collection, NE-PER Nuclear and Cytoplasmatic Extraction Reagents were used (Pierce Biotecnology, Waltham, MA, USA) following the protocol provided by the manufacturer. Protein concentration was determined with the BCA Protein Assay (Pierce Biotecnology, Waltham, MA, USA). The same amount of protein extract per condition was loaded onto the polyacrylamide gel. Once the run was completed, proteins were transferred to a nitrocellulose membrane using an iBlot 2 Dry Blotting System (Invitrogen, Waltham, MA, USA) and iBlot 2 NC Regular Stacks (Invitrogen, Waltham, MA, USA). Membranes were blocked 1 h at RT with 5% BSA, and incubated overnight at 4 °C with specific primary antibodies. Primary antibodies used were GAPDH (MAB347, MERK, Poole, Dorset, UK), p-JNK (sc-6245, Santa Cruz Biotechnology Inc., Dallas, TX, USA), p-PKC δ/θ (#9376, Cell Signaling Technology Inc., Danvers, MA, USA ), and β-catenin (ab6302, Abcam, Cambridge, UK).

Membranes were then washed with TBS-T and incubated 1 h with specific secondary antibodies: IRDye 680LT Goat anti-rabbit (926-68021, LI-COR, Lincoln, NE, USA) and IRDye 800CW Donkey anti-Mouse (926-32212, LI-COR, Lincoln, NE, USA). Membranes were revealed with the LI-COR Odyssey Imaging System, and membrane signals revealed were analyzed and quantified with the Image Studio 5.2 program (LI-COR, Lincoln, NE, USA).

### 2.11. In Vivo Experiments

All surgical procedures were performed under isoflurane anesthesia in sterile conditions as previously described [26]. Discoidal alginate scaffolds (4 mm in diameter and 1 mm thickness) were implanted subcutaneously in C57/BL6 12 weeks old female mice on both sides of the dorsal line, leaving at least 1 cm separation between them. Scaffolds were seeded with un-transduced mouse MSCs (mMSCs) or mMSCs transduced with the Control (Ctrl), *Smurf1*, *Sfrp1*, *Cby1*, and *Tob1* GapmeRs and their combinations following the transfection conditions and experimental procedures previously described for in situ transfection. Scaffolds with no cells were also used as the control. Scaffolds were incubated with the cells in the standard growing media used for those cells for a minimum of 20 min and a maximum of 24 h before implantation. A fourteen hours incubation was used in this occasion. Scaffolds used have a dose of 3 μg BMP2 as previously described [6]. All scaffolds carrying cells transfected with the different GapmeRs were surgically implanted in the same animal to avoid individual variations. As an example, three animals were implanted with all the control scaffolds plus one scaffold seeded with cells transfected with the *Smurf1* GapmeR, one scaffold seeded with cells transfected with the *Sfrp1* GapmeR and one scaffold with cells transfected with a combination of the two GapmeRs tested (*Smurf1*/*Sfrp1*). A total of nine animals were used for the procedure. Three different experimental groups including three animals per group were established in the experiment, each one carrying a different GapmeR combination. Each of the animals received six different scaffolds. Insertion of the scaffold was performed through a cut in the skin of approximately 0.5 cm. To close the incision, the skin was stapled. Twelve weeks after the procedure, animals were euthanized by CO_2_, implants were removed from the mice, and fixed in 10% formaldehyde, decalcified, and embedded in paraffin. Five micron sections were prepared for the different analyses.

All experiments performed in animals were reviewed and approved on August 2016 by the Institutional Bioethics and Animal Care Committee of the University of Cantabria (Project identification code 2015721, PI08/16) and the competent authority (Consejería de Agricultura y Ganadería de Cantabria, Santander, Spain).

### 2.12. Histological Analysis

The implants extracted from the mice were fixed in 10% formaldehyde for 6 h and decalcified in 20% EDTA in PBS (pH 7.4) for a week at 4 °C with two weekly changes of the descaling solution. Once decalcified, they were embedded in paraffin and 5 micron sections were obtained that were deparaffined and stained following standard protocols with topographic hematoxylin/eosin staining [25], and Masson-Goldner Trichrome [26] or Sirius Red histochemical stains [27] for collagen detection. For the indirect immunoenzymatic technique, the deparaffined and rehydrated sections in Tris buffered saline (TBS) (pH 7.4, 0.01 M Trizma base, 0.04 M Tris hydrochloride, 0.15 M NaCl) were subjected to antigen retrieval in citrate buffer (pH 6) for 5 min at 90 °C and subsequently blocked in 2% FBS in 0.2% TBS-Triton (blocking buffer). For the study of osteogenic differentiation and neovascularization, the sections were incubated with polyclonal anti-osteocalcin (OCN) antiserum (1/100) (Millipore) and polyclonal anti-CD34 antiserum (1/100) (Abyntek Biopharma, Derio, Spain ), respectively, in blocking buffer overnight at 4 °C. The sections were then washed with TBS and incubated with a donkey anti-rabbit IgG antibody conjugated with biotin (1/500) (Millipore, Poole, Dorset, UK) for 60 min and then with streptavidin-peroxidase (1/500) (Millipore, Poole, Dorset, UK) for 60 min. Peroxidase activity was demonstrated in 0.005% 3,3‘-diaminobenzidine (Sigma-Aldrich, Poole, Dorset, UK) and 0.01% hydrogen peroxide in Tris-HCl buffer (0.05 M, pH 7.6). The specificity of the immunostaining with both antisera was confirmed by replacing them with normal serum. Vascular density and OCN staining was evaluated using computer-based image analysis software (Leica Q-win V3 Pro-Image Analysis System). To measure vascular density, the CD34 positive staining blood vessels were counted in five different areas in implant sections for each sample and expressed in absolute value as the number of vessels per unit area (microscopic field). OCN staining was measured by applying a fixed threshold to select for positive staining within the implant region. Positive pixel areas were divided by the total surface size (mm^2^) of the implant. Values were normalized to those measured from Control GapmeR group and are reported as relative staining intensities.

### 2.13. Statistical Analysis

Error bars on graphs represent the standard error of the mean values. Depending on sample size, statistical significance was calculated using the Students’ t test (for *n* > 5) or the Mann–Whitney U test (for *n* = 3). To assay the bone formation in the mouse ectopic model, statistical analysis was performed with SPSS.25 software by means of a one-way analysis of variance (ANOVA) with a Tukey multiple comparison post-test. Significance was set at *p* < 0.05.

## 3. Results

### 3.1. Efficient Silencing of Osteogenic Inhibitors in MSCs Using LNA-ASOS

**Figure 1:**
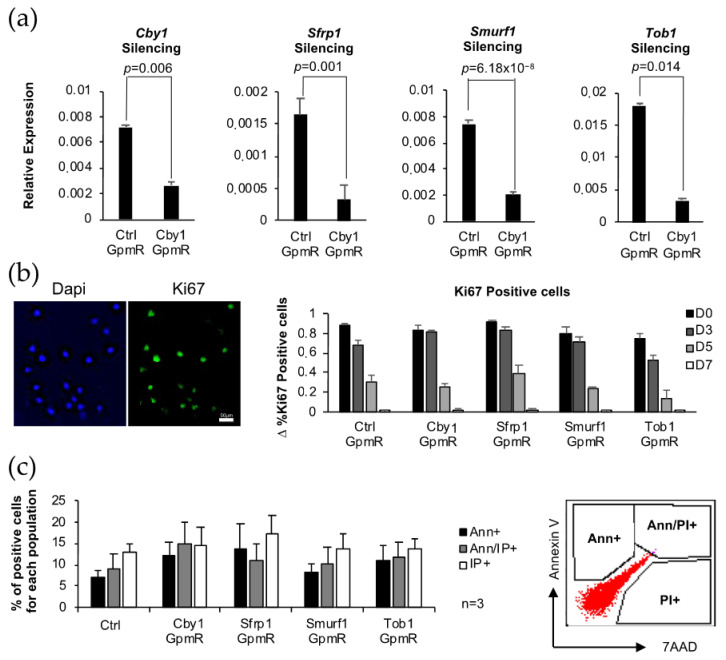

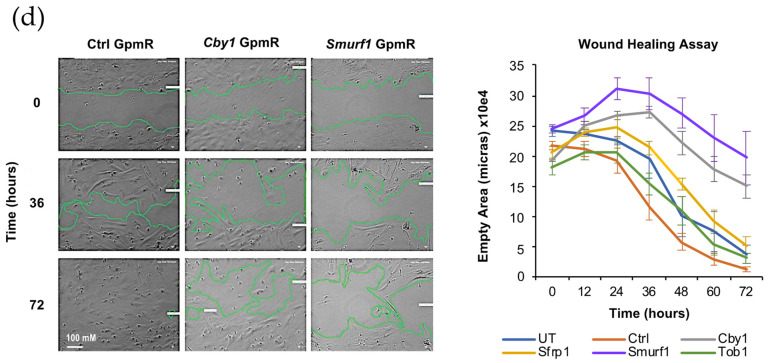
Analysis of proliferation, apoptosis, and migration of MSCs transfected with GapmeRs specific for the silencing of key osteogenic inhibitors. (**a**) Silencing of the different targeted genes in C3H10T1/2 MSCs using specific GapmeRs 48 h after transfection. Ctrl stands for Control GapmeR (*n* = 7). Error bars represent standard error of the mean values. (**b**) Representative picture of the immunofluorescence study of Ki67 expression in cells transfected with the different GapmeRs proliferating in normal culture media at different timepoints. Graph represents the increment of fluorescent cells referred to the previous day in each of the conditions tested. (**c**) Flow cytometry analysis of apoptosis using Annexin V and 7AAD. Average values of three different flow cytometry analysis are shown. Cells were stained with Annexin V (AnnV) and propidium iodide (PI) to detect early apoptosis (Ann+), late apoptosis (Ann/PI+), or dead cells (PI+). Percentage of positive cells for each population was normalized to cells transfected with the control GapmeR (Ctrl). Flow cytometry profile is shown to define populations. (**d**) Wound healing assay. Representative images of cells underperforming in the assay are shown from three independent experiments. Areas lacking cells are outlined. The graph shows the average values and the standard error of at least six different points along the wound measured for each of the samples from a representative experiment.

Our working hypothesis is that silencing of inhibitors of the BMP signaling pathway other than our previous target, *Smurf1*, and/or silencing of inhibitors of the Wnt/β-catenin signaling pathway, either alone or in combination, would result in a substantial increase in the MSCs osteogenic potential. In order to prove this, we first needed to check the efficiency of gene silencing induced by the different GapmeRs. We transfected a monolayer culture of the murine MSCs line C3H10T1/2 with GapmeRs specifically designed to silence each of the selected genes. The use of this cell line allowed us to reduce the number of animals used in the study, therefore complying with the principles of humane experimentation. As previously reported, transfection efficiency achieved by using this method was always over 90%, as shown by the control fluorescent GapmeR internalization measured by flow cytometry (data not shown). Expression analysis of each of the targeted genes 48 h after transfection showed that in all cases, gene silencing was highly efficient, achieving between 75 and 80% of silencing in all cases, compared to cells transfected with the control GapmeR (Figure 1a). This same transfection protocol was followed for the subsequent experiments, consistently obtaining similar silencing efficiencies. Gene silencing was always verified before every analysis. It is important to highlight that the silencing produced by the GapmeRs is only transient, and their effect declined over the following days. In fact, we could see that only 11 days after transfection, the expression of the targeted genes had increased by 50% compared to the first measure performed just 48 h after transfection leaving, at this time, an overall silencing of less than 30% for all the analyzed genes (Supplementary Figure S1).

### 3.2. Biosafety Evaluation of MSCs Expressing Low Levels of Key Osteogenic Inhibitors

An important problem associated with the alteration of the BMP and Wnt/β-catenin pathways is that those alterations have frequently been found linked to cancer progression and other diseases, highlighting the important role of these pathways in the control of cellular proliferation, invasion, metastasis, and cell death [28,29,30]. Due to these implications and, since our ultimate goal is the therapeutic use of these cells where the expression of key osteogenic inhibitors has been modified, it is crucial to first check their biosafety. To this end, we performed a series of tests aimed to analyze whether processes such as proliferation, migration, and apoptosis of the transfected cells have been altered in a way that could favor tumor formation and progression.

### 3.3. Effect of Silencing of the Target Genes on Non-Canonical Wnt Pathways

**Figure 2:**
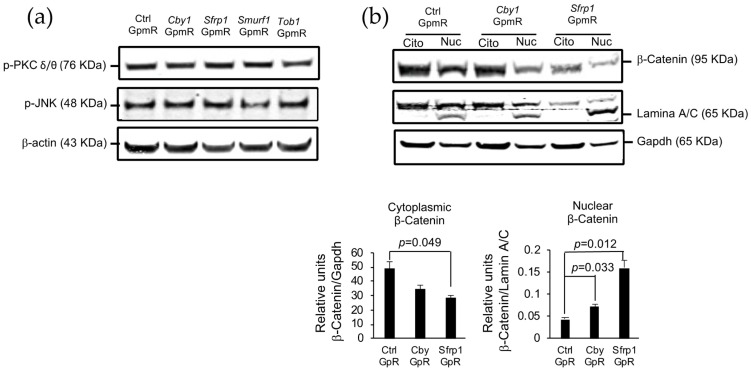
Effect of *Cby1* and *Sfrp1* silencing on canonical and non-canonical Wnt pathways. Each experiment was performed in duplicates. A representative experiment is shown. (**a**) Western blot of JNK and PKC phosphorylation used as markers of the Wnt/PCP and the Wnt/PKC pathway activation. Forty-eight hours after transfection with the different GapmeRs, osteogenic media was added for an additional 48 h before whole protein extracts were obtained. β-actin was used as a loading control. (**b**) Presence of the nuclear and cytoplasmic β-catenin was quantified in nuclear and cytoplasmic cell extracts. Lamin A/C and Gapdh were used for normalization of the nuclear and cytoplasmic signals, respectively.

Wnt proteins are able to activate the Wnt/β-catenin canonical pathway that leads to the translocation of β-catenin to the nucleus, but also other pathways that regulate calcium flux and cascades of kinases. One of them, the Wnt/PCP (Planar Cell Polarity) pathway results in the activation of the Jun-N terminal kinase (JNK), which activates c-JUN, the last transcription factor of the pathway. The other non-canonical Wnt pathway, also named the Wnt/PKC (Protein Kinase C) is a Ca^2+^ dependent pathway that leads to the release of the intracellular calcium, and thus to the activation of Ca^2+^ sensitive enzymes such as PKC [31]. Since in other cell types these other non-canonical pathways have been shown to be activated by the binding of *Sfrp1*, we wanted to check whether *Sfrp1* inhibition or the inhibition of *Cby1*, also acting in the canonical Wnt pathway, could significantly alter the activity of the Wnt/PCP and Wnt/JNK pathways in MSCs. We evaluated the phosphorylation of JNK as a marker of the Wnt/PCP pathway activation and the phosphorylation of PKC as a marker for the activation of the Wnt/PKC pathway by western blot in MSCs transfected with the different GapmeRs. We were not able to detect any increase in the phosphorylation of either PKC or JNK that would suggest an abnormal activation of the non-canonical Wnt pathways (Figure 2a).

In parallel, we also checked the presence of β-catenin in the nucleus and cytoplasm when *Cby1* and *Sfrp1* are silenced. Through this analysis, we could verify that upon silencing of those genes, there is a significant increase in the presence of β-catenin in the nucleus of the cells, confirming an activation of the Wnt/β-catenin pathway (Figure 2b).

### 3.4. In Vitro Assessment of Osteogenic Capacity of MSCs Where Expression of Anti-Osteogenic Genes Has Been Downregulated

**Figure 3:**
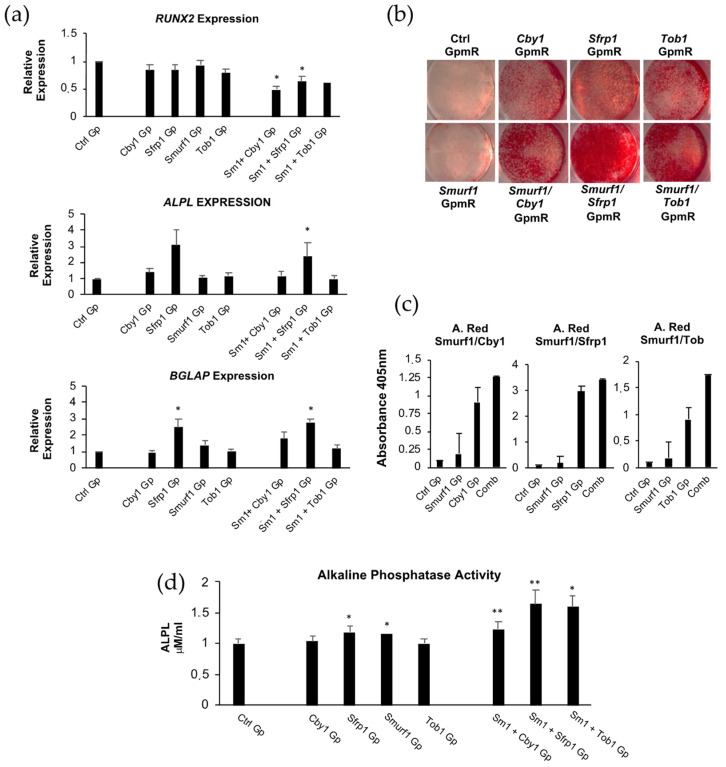
Osteogenic differentiation capacity of MSCs after the silencing of specific osteogenic inhibitors and their combinations. Sm1 stands for *Smurf1* in the GapmeR combinations. All analyses were performed on transfected cells that had undergone osteogenic differentiation for 11 days. All graphs represent average values of independently performed experiments. Error bars represent standard error of the mean values. For analysis performed on cells transfected with a single GapmeR (*n* = 7) and for those performed with cells transfected with GapmeR combinations (*n* = 5). (**a**) Expression of the osteoblastic differentiation driver *Runx2* and osteogenic markers *Alpl* and *Bglap*. (**b**) Alizarin Red staining of cells transfected with GapmeRs or GapmeRs combinations growing in osteogenic media. The picture shows a representative experiment performed at day 11 of differentiation. (**c**) Graphs shows quantification of the Alizarin Red staining at 405 nm. (**d**) Graph shows alkaline phosphatase activity measured in the different conditions. (* *p* ≤ 0.05; ** *p* ≤ 0.005).

Prior to testing the in vivo osteogenic potential of MSCs after the silencing of the targeted genes, we performed a series of analyses to evaluate this potential in vitro. Gene expression analysis of cells treated with the different GapmeRs, either individually or combined, growing in osteogenic media, showed important differences in the expression of key osteogenic markers (Figure 3a). Although in all the cases a downregulation of *Runx2* (Runt-related transcription factor 2), the main gene driving osteogenic differentiation, was detected, this downregulation was only significant in the case of cells where *Smurf1* and *Sfrp1* had been simultaneously silenced. This was also true for the combination of the *Smurf1* GapmeR with the *Cby1* GapmeR. The expression of the gene encoding alkaline phosphatase (*Alpl*) significantly increased when the *Smurf1* and *Sfrp1* GapmeRs were combined. This particular result correlates with that obtained by quantifying the alkaline phosphatase activity, which plays a critical role in mineralization (Figure 3d) where *Sfrp1* and *Smurf* seem to be the only genes able to produce a significant increase in this activity when individually silenced.

The expression of osteocalcin (*Bglap*), a gene expressed later during osteogenic differentiation, usually considered characteristic of the osteoblastic phenotype and a target of RUNX2, is significantly upregulated upon silencing of *Sfrp1* and when *Smurf1* is simultaneously silenced in these cells. It is noteworthy that MSCs treated with *Sfrp1*, *Tob1*, and *Cby1* GapmeRs displayed an increased ability to form a mineralized matrix when cultured in osteogenic differentiation media compared to those MSCs transfected with the control GapmeR (Figure 3b,c). The quantification of this mineralization showed that this process was particularly high in MSCs treated with *Sfrp1*, with a mineralization degree that was almost twice as high as the rest of the cells tested (Figure 3c). Please note the different scales used for representing each of the graphs in Figure 3c. In this case, the simultaneous silencing of *Smurf1* does not seem to substantially increase the mineralization.

These analyses of the osteogenic capacity were complemented with an analysis of the expression of the key adipogenic markers *Pparγ* (Peroxisome Proliferator Activated Receptor Gamma) and *Lpl* (Lipoprotein Lipase) at day 7 of adipogenic differentiation (Supplementary Figure S3). No significant differences were observed between the cells transfected with the Ctrl GapmeR and those transfected with the GapmeRs specific for the silencing of the different target genes and their combinations at that time point. However, the quantification of Oil Red, a dye that specifically stains lipids, showed a significant decrease in the amount of Oil Red in the MSCs simultaneously transfected with the *Smurf1/Sfrp1* GapmeR combination, suggesting a reduced adipogenic potential of those cells.

### 3.5. Assessment of Osteogenic Capacity of the Transfected MSCs In Vivo

To analyze the osteogenic capacity of cells where the different target genes and their combinations have been silenced, transfected cells were seeded in alginate scaffolds and incubated overnight for 14 h before they were implanted ectopically in C57/BL6 mice as previously described [6]. This ectopic model would prevent MSCs mobilized from the surrounding tissue from seeding the scaffold, which would allow us to specifically evaluate the contribution of the transplanted cells to bone tissue formation in the scaffold. To comply with current guidelines on the use of experimental animals and in order to reduce the number of mice used, we performed several implants into a single mouse and compared different implant conditions [32,33]. This arrangement is suited for experiments using inbred mice, since the use of these animals highly reduces inter-individual variability. A total of nine mice were used for this experiment, each of them carrying six scaffolds implanted subcutaneously. Our experimental design aimed to have all controls and combinations tested in the same animal to avoid minor inter-individual variations. Each animal carried three control scaffolds and three other scaffolds. As controls, we used an empty scaffold, a scaffold seeded with untreated MSCs, and a scaffold seeded with cells transfected with the Ctrl GapmeR. Of the remaining three scaffolds implanted in the animal, two were always seeded with cells treated with individual GapmeRs (*Smurf1* and the other tested target gene), and the remaining one was seeded with cells treated with the combination of both GapmeRs. To further clarify this point, three mice were used to test the bone regeneration potential of cells treated either with the *Smurf1* GapmeR, the combination of the *Smurf1*/*Cby1* GapmeRs or the *Cby1* GapmeR alone. Another set of three mice will carry scaffolds seeded with cells transfected with either *Smurf1*, *Smurf1/Tob1*, or *Tob1* GapmeRs. Finally, the remaining three mice were used to test in vivo the effect of the MSCs transfected with either the combination of the *Smurf1*/*Sfrp1* GapmeRs or with each of those GapmeRs individually.

**Figure 4:**
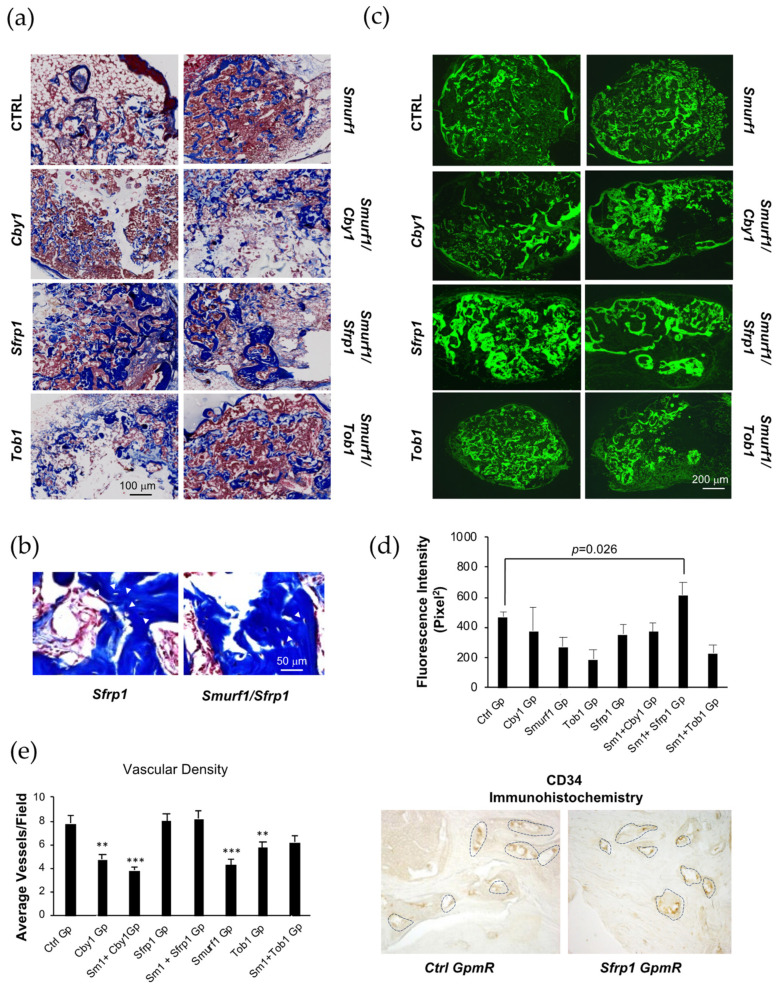
In vivo analysis of the pro-osteogenic effect of the silencing of key osteogenic inhibitors in MSCs. Pictures show histological analysis of sections obtained from representative decalcified implants. All implants were isolated from the same individual. (**a**) Masson–Goldner staining of subdermal implants in mice, showing mineralized matrix. (**b**) Representative images of Masson–Goldner staining at higher magnification. White arrowheads indicate osteocyte-like cells surrounded by lacunae and immersed in the mineralized matrix. (**c**) Histological sections observed under green, fluorescent light show the typical autofluorescence of collagen fibers in the different samples analyzed. (**d**) Bar graph shows quantification of fluorescence signal intensity in the different histological samples performed using ImageJ. Sm1 stands for *Smurf1*. One implant of each type was quantified. Values correspond to the average of at least six different areas measured in each of the implants. Error bars represent standard error of the mean values. Sm1 stands for *Smurf1* in the Gapmer combinations. (**e**) Immunohistochemistry of vascular marker CD34. Microvascular density was determined by counts of CD34-positive staining blood vessels. Sm1 stands for *Smurf1* in the Gapmer combinations. The graph shows the average values and the standard error of at least five different areas analyzed for each sample. Microscopic appearance of CD34-positive staining blood vessels is shown on the right. Stained microvessels are outlined (** *p* ≤ 0.005; *** *p* ≤ 0.0005).

After 12 weeks, the implants were surgically removed, and the extent of new bone formation was analyzed by histological techniques. An average of three scaffolds for condition were analyzed in these experiments. Although bone-type matrix was present to some extent in all scaffolds carrying MSCs, similar to what we have described in previous publications [6], this was particularly abundant in those scaffolds seeded with cells transfected with the *Sfrp1* and the *Sfrp1*/*Smurf1* GapmeR combination, as shown by extensive areas of collagen stained in a dark blue color with the Masson–Goldner technique (Figure 4a). In these particular samples, higher magnifications showed an abundance of cells embedded within the matrix and surrounded by an empty region resembling osteocytes within osteocytic lacunae (Figure 4b). Scaffolds seeded with cells where either *Sfrp1* alone or in combination with *Smurf1* had been silenced also showed an increased autofluorescence, typical of bone, under green, fluorescent light (Figure 4c,d) compared to the other conditions analyzed. Sections of the different scaffolds were also stained with Sirius Red to observe the disposition of collagen fibers under polarized light. In Supplementary Figure S4a, it is possible to observe clear bright areas corresponding to the birefringence of collagen fibers structured in an antiparallel disposition, typical of organized lamellar bone. In addition, immunohistochemistry analysis using an antibody that recognized osteocalcin showed that all samples were immunoreactive for this protein (Supplementary Figure S4b) and all except *Cby1* showed significant increases in OCN presence compared to the control scaffold.

Since a successful bone formation is tightly connected to vessel infiltration, we quantified the presence of blood vessels in all the implanted scaffolds through immunohistochemistry using a CD34 antibody. Our results showed that while the other conditions tested led to a significant reduction in the density of blood vessels, in those scaffolds that had been seeded with *Sfrp1* or the *Sfrp1*/*Smurf1* silenced MSCs, the blood vessel density was maintained within a normal range when compared to that of the control (Figure 4e).

### 3.6. In Vitro Assessment of the Effect of Sfrp1 Silencing on the Osteogenic Capacity of hMSCs from Osteoporotic Patients

Since MSCs from osteoporotic individuals (hMSC-OP) have been shown to have a reduced osteogenic capacity, we wanted to test if the silencing of *Sfrp1* could also improve the osteogenic potential of those cells.

We designed GapmeRs to specifically silence the human *SFRP1* gene and transfected human MSCs isolated from osteoporotic patients using previously established protocols. After transfection with the correspondent GapmeRs, cells were grown in an osteogenic induction media for 21 days. In parallel, we also grew hMSCs-OP in the presence of 10 ng/mL of BMP-2 for comparison. As we have previously shown, this BMP-2 concentration is enough to induce osteogenic differentiation in vitro in these cells [6].

**Figure 5:**
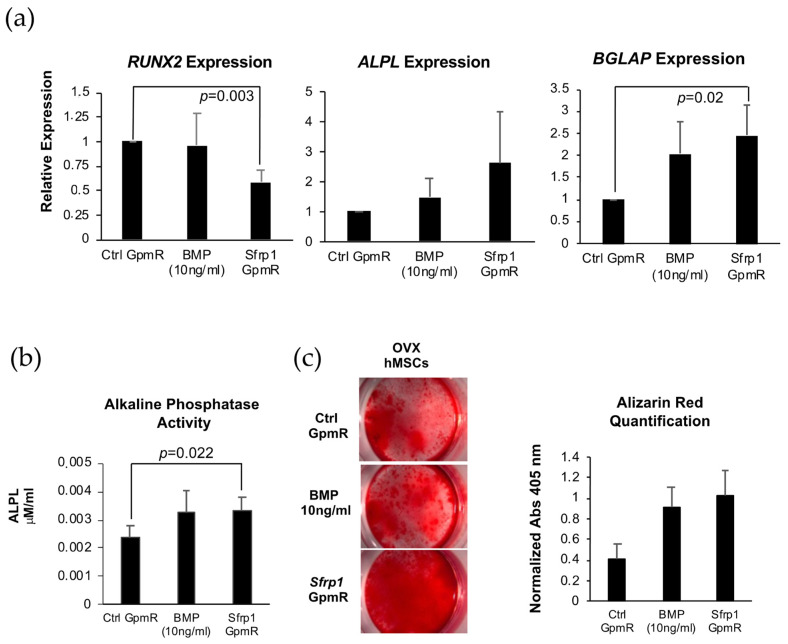
Effect of *SFRP1* silencing on the osteogenic potential of human MSCs from osteoporotic patients. All analyses were performed at 21 days of osteogenic differentiation. (**a**) Semi-quantitative PCRs showing the relative expression levels of different osteogenic markers (*RUNXx2*, *ALPL*, and *BGLAP*) in hMSCs from osteoporotic patients (MSC-OP) transfected with a control GapmeR (Ctrl) and a GapmeR for the silencing of *SFRP1* expression. The expression of the analyzed markers in the presence of 10 ng/mL of BMP-2 was used as a reference of the effectiveness of *SFRP1* silencing. Values reflect averages of five different samples (*n* = 5). Technical triplicates were also performed with each individual sample. (**b**) Graph showing the ALPL activity in MSC-OP under the different experimental conditions analyzed. Graph shows average values of five independent samples. (**c**) Osteogenic differentiation of the same hMSCs-OP transfected with the *SFRP1* GapmeR or differentiated in the presence of 10 ng/mL BMP-2 as revealed by Alizarin Red staining. Results from one representative sample are shown in the pictures. Bar graph represents quantification of the staining. In all graphs, bars represent standard error of the mean values. *p*-values are indicated.

We then assessed the expression of key osteogenic markers in all conditions. In hMSCs-OP transfected with the *SFRP1* GapmeR, we detected a significant downregulation of *RUNX2* expression, suggestive of an accelerated osteogenesis. The analysis of the late osteogenic marker *BGLAP* corroborates a comparable upregulation of the expression of this gene in hMSCs-OP where *SFRP1* had been silenced (Figure 5a). Although we could not find a significant change in the expression of the *ALPL* gene, we did find a significant upregulation in the ALPL activity in cells where *SFRP1* had been silenced (Figure 5b). These results were further supported by an increased calcium deposition in the tested conditions compared to the control at day 21 of osteogenic differentiation, as can be seen in Figure 5c, highlighting the pro-osteogenic potential of *SFRP1* silencing in hMSCs-OP.

## 4. Discussion

The numbers and potency of MSCs are key to maintaining normal bone physiology, therefore, modifications aimed to increase MSCs osteogenic potential could result in being highly useful to develop efficient therapies to promote bone regeneration. Modulating the two main osteogenic pathways, the BMP and the Wnt/β-catenin signaling pathways, in order to increase or extend their activity seems a reasonable approach to achieve this goal. We have previously shown that transiently silencing *Smurf1*, one of the main inhibitors of the BMP pathway, using LNA-ASOs, leads to a significant increase in bone formation, even in the presence of low BMP doses [6]. This modification of MSCs allowed us to increase bone formation by at least 30%. In this current work, we set out to further improve this system in order to achieve superior bone regeneration by targeting other inhibitors of the two main osteogenic pathways.

The relevance of the canonical Wnt signaling pathway in bone formation is underscored by the direct relation of mutations that activate or inactivate this route with sclerosteosis and osteoporosis, respectively. Different approaches have been designed to stimulate this osteogenic pathway, either through the upregulation of key players of the signaling cascade or through the inhibition of endogenous antagonists. A good example of the latest is the recently, FDA approved humanized anti-sclerostin monoclonal antibody Romosozumab (Amgen, Thousand Oaks, CA, USA), used for the treatment of osteoporosis. The suitability of other members of this route as potential targets for bone regeneration techniques are currently under investigation. Extracellular modulators of the Wnt/β-catenin pathway also seem to have a key role in bone regeneration. One of these modulators, *Sfrp1*, acts as an inhibitor of the Wnt canonical pathway by binding to Wnt ligands and preventing the activation of this pathway. Although the manipulation of the Wnt signaling cascade could bring several potential benefits, as shown by the use of Romosozumab, alterations of this route have also been linked to various pathologies including cancer, and thus, these manipulations should be performed with extreme caution [34]. While *Sfrp1* loss of expression in other cell types has been linked to abnormal cell proliferation, we did not detect significant changes in either cell proliferation or apoptosis in MSCs upon *Sfrp1* silencing. Another possible problem that could be encountered after *Sfrp1* silencing is the increase in cell migration and invasion related to an induction in a partial epithelial-mesenchymal transition [35]. This has been reported in a non-malignant cell line (mammary epithelial cell line) and was reflected by an increased motility in a scratch wound assay noticeable after just 8 h incubation. Using a similar assay, we did not find either a significant increase in the motility of MSCs transfected with an LNA-ASO specific for *Sfrp1* after 72 h of incubation. Therefore, our results would indicate that the transient silencing of this gene in MSCs does not seem to produce an increase in the tumorigenic potential of the transfected MSCs. This is particularly important since these cells are ultimately intended for therapeutic use. It is noteworthy that although, as previously shown by us, *Smurf1* silencing using LNA-ASOs led to a significant increase in the osteogenic potential of MSCs, the wound healing experiments showed that these MSCs where *Smurf1* or *Cby1* have been silenced show a slightly reduced migration capacity, something that could have a negative effect on fracture healing, particularly in aged or osteoporotic patients in which MSCs already feature a reduced migration ability [36,37]. Besides, this reduced migration could also limit the application of these cells in some treatments where migration of exogenous cells to the site of damage is required.

Since the canonical Wnt signaling pathway seems to crosstalk with other Wnt pathways, to be able to use *Sfrp1* silencing in MSCs, either alone or in combination with other inhibitors of osteogenic pathways, as a strategy to safely potentiate bone regeneration, the effect of its silencing in other signaling pathways should also be assessed. As expected, upon *Sfrp1* silencing, we did detect a significant increase in the nuclear β-catenin, indicating an activation of the Wnt/β-catenin signaling pathway, however, no apparent changes were detected in the selected mediators of the other two Wnt pathways that activate diverse biological functions including proliferation, apoptosis, and invasion. The same would apply to the case of *Cby1*, the other Wnt/β-catenin inhibitor silenced in this study. These results would agree with the outcome of our Ki67, MTT, and Annexin V analyses, which showed no alteration of those processes upon silencing of *Sfrp1*, or any of the other targeted genes, in MSCs. Overall, we could not find any indication that transiently silencing *Sfrp1* in MSCs using LNA-ASOs would be linked to any biosafety concern.

Of all the inhibitors and inhibitor combinations tested, our in vitro results clearly show that *Sfrp1* silencing has the more marked effect in osteogenesis, overcoming those of our previous selected target, *Smurf1*, and the other inhibitors tested in this work. RUNX2 is a master regulator of osteoblastic differentiation and plays a critical role in the early stages of this process, however, its expression needs to be downregulated to allow for the expression of later genes and terminal differentiation of osteoblasts [38]. We observed that MSCs where *Sfrp1* expression had been silenced in combination with *Smurf1* and *Cby1* showed a significant downregulation of *Runx2* expression at 11 days of differentiation. On the other hand, *Sfrp1* silencing either alone or in combination with *Smurf1* also led to a clear increase in the *Alpl* expression, although this was statistically significant only for the *Smurf1*/*Sfrp1* GapmeR combination. Only the combination of *Sfrp1* with *Smurf1* led to a significant up-regulation of the later osteogenic marker *Bglap*. It is important to note that the earlier downregulation of *Runx2* and upregulation of late osteogenic genes seen with the *Smurf1*/*Sfrp1* GapmeR combination would be indicative of an accelerated osteogenesis. The upregulation of *Alpl* at the gene expression level was also in agreement with the quantified ALPL activity. In this case, *Sfrp1* and *Smurf1* were the only GapmeRs able to significantly increase this activity when tested individually. Altogether, these data suggest an increased osteogenic potential of cells treated with the *Sfrp1* GapmeR alone or in combination with the *Smurf1* GapmeR. An enhanced mineralization upon *Sfrp1* silencing, as measured by Alizarin Red staining, further supports this notion. Despite the efficient bone formation elicited by the silencing of *Smurf1* in vivo [6], neither significant changes in *Alpl* expression nor increased mineralization were detected in vitro when this gene was silenced. It is important to highlight, though, that, except for *Smurf1*, the silencing of the other anti-osteogenic genes also led to an increase in mineralization compared to the control. Interestingly, when cells were grown in adipogenic media, we observed a decrease in viability of cells transfected with *Smurf1*/*Sfrp1* or with *Smurf1*/*Tob1* GapmeRs, although the initial decrease in proliferation seems to be recovered later on. Since this initial phase would coincide with the clonal expansion of adipogenic progenitors prior to proliferation, this might reflect a reduced adipogenic capacity. This idea would be further supported by a reduced Oil Red staining of the cells transfected with *Sfrp1* and *Smurf1* growing under adipogenic conditions, as expected by the upregulation of the canonical Wnt/β-catenin pathway [39].

The in vitro results were further validated in our in vivo ectopic model where *Sfrp1* silencing also performed significantly better than the rest of the targeted genes. This ectopic system allowed us to specifically evaluate the contribution of the implanted cells to bone formation. The analysis of the cell-loaded scaffolds showed that all types of MSCs transplanted were able to form bone tissue to a greater or a lesser extent after 12 weeks, however, those cells where *Sfrp1* had been silenced, either alone or together with *Smurf1* were able to produce a significantly higher amount of bone matrix in vivo, as reflected by the larger areas of collagen fibers shown by Masson–Goldner trichromic and Sirius Red stainings and by the analysis of the samples under fluorescent light. Immunohistochemistry analysis to detect the presence of osteocalcin (OCN), encoded by the *Bglap* gene in the different scaffolds, showed a significantly higher signal than the control in all samples, except for *Cby1* when compared to the control sample (Supplementary Figure S4b). Since OCN is produced by osteoblasts and is used as a late marker in the process of bone formation, these results suggest the presence of a more mature bone matrix in those samples. Importantly, unlike the rest of the samples tested, the scaffolds seeded with MSCs where *Sfrp1* had been silenced reached a degree of angiogenesis similar to that of the MSCs transfected with the Ctrl GapmeR. Since normal angiogenesis is key for correct bone healing [40], this further supports the choice of *Sfrp1* as the best target to achieve efficient bone regeneration through the LNA-ASO mediated silencing of osteogenesis inhibitors in MSCs. It is noteworthy that although the simultaneous silencing of *Smurf1* and *Sfrp1* seems to have, in vitro, an additive effect in the expression of osteogenic markers as well as in the alkaline phosphatase activity, no significant improvement in bone formation was detected upon simultaneous silencing of *Smurf1* and *Sfrp1* in our in vivo experiments, suggesting that the sole silencing of *Sfrp1* would suffice to efficiently prime MSCs in vivo toward the osteogenic differentiation path.

It has been suggested that the anabolic effect of the parathyroid hormone (PTH), an FDA approved osteo-anabolic drug commonly used for osteoporosis treatment, requires *Sfrp1* downregulation to stimulate Wnt signaling and osteoblastogenesis [41]. Thus, *Sfrp1* silencing would have a similar effect to that of the PTH but would lack the detrimental side effects of this drug that highly limit the length of the treatment [42], something extremely important due to the chronic character of that pathology. Furthermore, our results using hMSC-OP show that *Sfrp1* silencing in those cells can achieve a similar or even greater osteogenic induction than that obtained through BMP-2 stimulation, therefore, the use of *Sfrp1* inhibition would have the additional advantage of avoiding the need of concomitantly administrating BMP-2 in bone regeneration therapies based on MSC transplantation and thus, of avoiding the unwanted side effects linked to the use of this protein in the clinic.

In the last few years, *Sfrp1* has been the focus of several studies related to bone regeneration. In fact, reports in mice where this gene has been inactivated have shown that fracture healing is potentiated in the absence of this gene [20]. This, and other works confirming the importance of *Sfrp1* in osteogenesis, have led to the development of different approaches aimed to achieve a safe *Sfrp1* inhibition that could be used for bone regeneration therapies. One study showed that diphenyl sulfone sulfonamide, a small-molecule inhibitor of *Sfrp1* that disrupts the interaction between Wnt and *Sfrp1*, is able to stimulate bone formation ex vivo [43]. More recently, the discovery that different miRNAs such as miR542-3p and miR144 have a positive effect on osteogenesis through *Sfrp1* downregulation [44,45] has opened the possibility of using these molecules as therapeutic agents to treat bone loss associated diseases. However, miRNA activity is pleiotropic, with a single miRNA repressing numerous targets, which in many cases are not even completely identified. This lack of specificity would also apply to drugs such as the aforementioned sulfone sulfonamide. Thus, in therapies based on the use of drugs or miRNAs mimics, it might be challenging to delineate all the possible effects in cells other than MSCs. This would be an obvious concern from the biosafety point of view.

Due to its ability to selectively modulate gene expression and the fact that they are a clinically safe method, LNA-ASOs have countless clinical applications. This is highlighted by the increasing number of oligonucleotide-based drugs gaining approval [46]. Despite the promising results of these molecules, the application of LNA-ASOs in the clinic has been hindered until recently by the difficulties in achieving efficient delivery to target organs. This would be particularly important in the case of *Sfrp1*, since it has the ability to, depending on the cellular context, act as an agonist or as an antagonist of the Wnt signaling pathway [47]. In this work, we have shown that the transient silencing of *Sfrp1* using these molecules is enough to efficiently prime MSCs toward the osteogenic route and favor an efficient bone matrix formation, even in osteoporotic MSCs where this osteogenic capacity is already limited. Treatment of a chronic pathology such as osteoporosis needs a very stringent biosafety profile. Although we have found no alteration of cell proliferation, apoptosis, or migration upon *Sfrp1* silencing in vitro, given the known involvement of Wnt signaling in certain types of cancer, translation of this method to the clinic will require further molecular analysis such as analysis of important tumor suppressor genes (p53), karyotyping, etc. and extensive in vivo pre-clinical studies to ensure long-term safety of the treatment. Additionally, to increase the biosafety of treatments involving Wnt modulators, bone should be targeted as specifically as possible to prevent systemic stimulation of the pathway. Combination of GapmeRs specifically designed for *Sfrp1* silencing with one of the targeted delivery methods that are currently being developed would open the possibility of specifically silencing this gene in bone marrow MSCs and thus safely undertake the treatment of systemic bone loss such as that associated with osteoporosis.

## Supplementary Materials

The following are available online at https://www.mdpi.com/article/10.3390/pharmaceutics13081277/s1, Figure S1: Gene silencing efficiency 11 days after transfection with the correspondent GapmeRs, Figure S2: MTT analysis of proliferative capacity after transfection with the GapmeRs silencing different genes and the correspondent combinations, Figure S3: Adipogenic differentiation capacity of MSCs after the silencing of specific osteogenic inhibitors and their combinations, Figure S4: In vivo analysis of the pro-osteogenic effect of the silencing of key osteogenic inhibitors in MSCs.

## References

[1] Zigdon-Giladi H., Rudich U., Michaeli-Geller G., Evron A. (2015). Recent advances in bone regeneration using adult stem cells. World J. Stem Cells.

[2] He X., Dziak R., Yuan X., Mao K., Genco R., Swihart M., Sarkar D., Li C., Wang C., Lu L. (2013). BMP2 genetically engineered MSCs and EPCs promote vascularized bone regeneration in rat critical-sized calvarial bone defects. PLoS ONE.

[3] Fierro F.A., Kalomoiris S., Sondergaard C.S., Nolta J.A. (2011). Effects on proliferation and differentiation of multipotent bone marrow stromal cells engineered to express growth factors for combined cell and gene therapy. Stem Cells.

[4] Kim D., Cho S.W., Her S.J., Yang J.Y., Kim S.W., Kim S.Y., Shin C.S. (2006). Retrovirus-mediated gene transfer of receptor activator of nuclear factor-kappaB-Fc prevents bone loss in ovariectomized mice. Stem Cells.

[5] Rodriguez-Evora M., Garcia-Pizarro E., del Rosario C., Perez-Lopez J., Reyes R., Delgado A., Rodriguez-Rey J.C., Evora C. (2014). Smurf1 knocked-down, mesenchymal stem cells and BMP-2 in an electrospun system for bone regeneration. Biomacromolecules.

[6] Garcia-Garcia P., Ruiz M., Reyes R., Delgado A., Evora C., Riancho J.A., Rodriguez-Rey J.C., Perez-Campo F.M. (2019). Smurf1 silencing using a LNA-ASOs/Lipid nanoparticle system to promote bone regeneration. Stem Cells Transl. Med..

[7] Del Real A., Perez-Campo F.M., Fernandez A.F., Sanudo C., Ibarbia C.G., Perez-Nunez M.I., Criekinge W.V., Braspenning M., Alonso M.A., Fraga M.F. (2017). Differential analysis of genome-wide methylation and gene expression in mesenchymal stem cells of patients with fractures and osteoarthritis. Epigenetics Off. J. DNA Methylation Soc..

[8] Benglis D., Wang M.Y., Levi A.D. (2008). A comprehensive review of the safety profile of bone morphogenetic protein in spine surgery. Neurosurgery.

[9] Carragee E.J., Hurwitz E.L., Weiner B.K. (2011). A critical review of recombinant human bone morphogenetic protein-2 trials in spinal surgery: Emerging safety concerns and lessons learned. Spine J. Off. J. N. Am. Spine Soc..

[10] Dmitriev A.E., Lehman R.A., Symes A.J. (2011). Bone morphogenetic protein-2 and spinal arthrodesis: The basic science perspective on protein interaction with the nervous system. Spine J. Off. J. N. Am. Spine Soc..

[11] Even J., Eskander M., Kang J. (2012). Bone morphogenetic protein in spine surgery: Current and future uses. J. Am. Acad. Orthop. Surg..

[12] Hsu W.K., Wang J.C. (2008). The use of bone morphogenetic protein in spine fusion. Spine J. Off. J. N. Am. Spine Soc..

[13] Jeon E.J., Lee K.Y., Choi N.S., Lee M.H., Kim H.N., Jin Y.H., Ryoo H.M., Choi J.Y., Yoshida M., Nishino N. (2006). Bone morphogenetic protein-2 stimulates Runx2 acetylation. J. Biol. Chem..

[14] Singh K., Dumonski M., Stanley T., Ponnappan R., Phillips F.M. (2011). Repeat use of human recombinant bone morphogenetic protein-2 for second level lumbar arthrodesis. Spine.

[15] Usui M., Yoshida Y., Tsuji K., Oikawa K., Miyazono K., Ishikawa I., Yamamoto T., Nifuji A., Noda M. (2004). Tob deficiency superenhances osteoblastic activity after ovariectomy to block estrogen deficiency-induced osteoporosis. Proc. Natl. Acad. Sci. USA.

[16] Boudin E., Fijalkowski I., Piters E., Van Hul W. (2013). The role of extracellular modulators of canonical Wnt signaling in bone metabolism and diseases. Semin. Arthritis Rheum..

[17] Gaur T., Lengner C.J., Hovhannisyan H., Bhat R.A., Bodine P.V., Komm B.S., Javed A., van Wijnen A.J., Stein J.L., Stein G.S. (2005). Canonical WNT signaling promotes osteogenesis by directly stimulating Runx2 gene expression. J. Biol. Chem..

[18] Baron R., Kneissel M. (2013). WNT signaling in bone homeostasis and disease: From human mutations to treatments. Nat. Med..

[19] Kawano Y., Kypta R. (2003). Secreted antagonists of the Wnt signalling pathway. J. Cell Sci..

[20] Gaur T., Wixted J.J., Hussain S., O’Connell S.L., Morgan E.F., Ayers D.C., Komm B.S., Bodine P.V., Stein G.S., Lian J.B. (2009). Secreted frizzled related protein 1 is a target to improve fracture healing. J. Cell Physiol..

[21] Rodriguez J.P., Montecinos L., Rios S., Reyes P., Martinez J. (2000). Mesenchymal stem cells from osteoporotic patients produce a type I collagen-deficient extracellular matrix favoring adipogenic differentiation. J. Cell Biochem..

[22] Takemaru K., Yamaguchi S., Lee Y.S., Zhang Y., Carthew R.W., Moon R.T. (2003). Chibby, a nuclear beta-catenin-associated antagonist of the Wnt/Wingless pathway. Nature.

[23] Argintar E., Edwards S., Delahay J. (2011). Bone morphogenetic proteins in orthopaedic trauma surgery. Injury.

[24] Gregory C.A., Gunn W.G., Peister A., Prockop D.J. (2004). An Alizarin red-based assay of mineralization by adherent cells in culture: Comparison with cetylpyridinium chloride extraction. Anal. Biochem..

[25] Kraus N.A., Ehebauer F., Zapp B., Rudolphi B., Kraus B.J., Kraus D. (2016). Quantitative assessment of adipocyte differentiation in cell culture. Adipocyte.

[26] Rodriguez-Evora M., Delgado A., Reyes R., Hernandez-Daranas A., Soriano I., San Roman J., Evora C. (2013). Osteogenic effect of local, long versus short term BMP-2 delivery from a novel SPU-PLGA-betaTCP concentric system in a critical size defect in rats. Eur. J. Pharm. Sci..

[27] Cowan C.M., Shi Y.Y., Aalami O.O., Chou Y.F., Mari C., Thomas R., Quarto N., Contag C.H., Wu B., Longaker M.T. (2004). Adipose-derived adult stromal cells heal critical-size mouse calvarial defects. Nat. Biotechnol..

[28] Kim I.Y., Lee D.H., Ahn H.J., Tokunaga H., Song W., Devereaux L.M., Jin D., Sampath T.K., Morton R.A. (2000). Expression of bone morphogenetic protein receptors type-IA, -IB and -II correlates with tumor grade in human prostate cancer tissues. Cancer Res..

[29] Shepherd T.G., Nachtigal M.W. (2003). Identification of a putative autocrine bone morphogenetic protein-signaling pathway in human ovarian surface epithelium and ovarian cancer cells. Endocrinology.

[30] Tarapore R.S., Siddiqui I.A., Mukhtar H. (2012). Modulation of Wnt/beta-catenin signaling pathway by bioactive food components. Carcinogenesis.

[31] Niehrs C. (2012). The complex world of WNT receptor signalling. Nat. Rev. Mol. Cell Biol..

[32] Cengiz I.F., Pereira H., Espregueira-Mendes J., Kwon I.K., Reis R.L., Oliveira J.M. (2019). Suturable regenerated silk fibroin scaffold reinforced with 3D-printed polycaprolactone mesh: Biomechanical performance and subcutaneous implantation. J. Mater. Science. Mater. Med..

[33] Lopez-Delgado L., Del Real A., Sanudo C., Garcia-Ibarbia C., Laguna E., Menendez G., Garcia-Montesinos B., Santurtun A., Merino J., Perez-Nunez M.I. (2021). Osteogenic capacity of mesenchymal stem cells from patients with osteoporotic hip fractures in vivo. Connect. Tissue Res..

[34] Van Camp J.K., Beckers S., Zegers D., Van Hul W. (2014). Wnt signaling and the control of human stem cell fate. Stem Cell Rev. Rep..

[35] Gauger K.J., Hugh J.M., Troester M.A., Schneider S.S. (2009). Down-regulation of sfrp1 in a mammary epithelial cell line promotes the development of a cd44high/cd24low population which is invasive and resistant to anoikis. Cancer Cell Int..

[36] Haasters F., Docheva D., Gassner C., Popov C., Bocker W., Mutschler W., Schieker M., Prall W.C. (2014). Mesenchymal stem cells from osteoporotic patients reveal reduced migration and invasion upon stimulation with BMP-2 or BMP-7. Biochem. Biophys. Res. Commun..

[37] Bustos M.L., Huleihel L., Kapetanaki M.G., Lino-Cardenas C.L., Mroz L., Ellis B.M., McVerry B.J., Richards T.J., Kaminski N., Cerdenes N. (2014). Aging mesenchymal stem cells fail to protect because of impaired migration and antiinflammatory response. Am. J. Respir. Crit. Care Med..

[38] Bruderer M., Richards R.G., Alini M., Stoddart M.J. (2014). Role and regulation of RUNX2 in osteogenesis. Eur. Cells Mater..

[39] Kawai M., Mushiake S., Bessho K., Murakami M., Namba N., Kokubu C., Michigami T., Ozono K. (2007). Wnt/Lrp/beta-catenin signaling suppresses adipogenesis by inhibiting mutual activation of PPARgamma and C/EBPalpha. Biochem. Biophys. Res. Commun..

[40] Sivaraj K.K., Adams R.H. (2016). Blood vessel formation and function in bone. Development.

[41] Yao W., Cheng Z., Shahnazari M., Dai W., Johnson M.L., Lane N.E. (2010). Overexpression of secreted frizzled-related protein 1 inhibits bone formation and attenuates parathyroid hormone bone anabolic effects. J. Bone Miner. Res. Off. J. Am. Soc. Bone Miner. Res..

[42] Deng J., Feng Z., Li Y., Pan T., Li Q., Zhao C. (2018). Efficacy and safety of recombinant human parathyroid hormone (1–34) are similar to those of alendronate in the treatment of postmenopausal osteoporosis. Medicine.

[43] Moore W.J., Kern J.C., Bhat R., Commons T.J., Fukayama S., Goljer I., Krishnamurthy G., Magolda R.L., Nogle L., Pitts K. (2009). Modulation of Wnt signaling through inhibition of secreted frizzled-related protein I (sFRP-1) with N-substituted piperidinyl diphenylsulfonyl sulfonamides. J. Med. Chem..

[44] Zhang X., Zhu Y., Zhang C., Liu J., Sun T., Li D., Na Q., Xian C.J., Wang L., Teng Z. (2018). miR-542-3p prevents ovariectomy-induced osteoporosis in rats via targeting SFRP1. J. Cell Physiol..

[45] Tang L., Lu W., Huang J., Tang X., Zhang H., Liu S. (2019). miR144 promotes the proliferation and differentiation of bone mesenchymal stem cells by downregulating the expression of SFRP1. Mol. Med. Rep..

[46] Roberts T.C., Langer R., Wood M.J.A. (2020). Advances in oligonucleotide drug delivery. Nat. Rev. Drug Discov..

[47] Hoeppner L.H., Secreto F.J., Westendorf J.J. (2009). Wnt signaling as a therapeutic target for bone diseases. Expert Opin. Ther. Targets.

