# Supplementary material for: Effective Osteogenic Priming of Mesenchymal Stem Cells through LNA-ASOs-Mediated Sfrp1 Gene Silencing
Source: Pharmaceutics. 2021 Aug 17;13(8):1277. doi: 10.3390/pharmaceutics13081277 (PMC8398380; doi:10.3390/pharmaceutics13081277)
Supplement: Supplementary file 1 [file pharmaceutics-13-01277-s001.zip › pharmaceutics-1317061-supplementary.pdf]

pharmaceutics

MDPI

Supplementary Material: Effective Osteogenic Priming of Mesenchymal Stem Cells through LNA-ASOs-Mediated *Sfrp1* Gene Silencing

Daniel García-Sánchez, Alberto González-González, Patricia García-García, Ricardo Reyes, María Isabel Pérez-Núñez, José A. Riancho, Carmen Évora, José Carlos Rodríguez-Rey and Flor M. Pérez-Campo

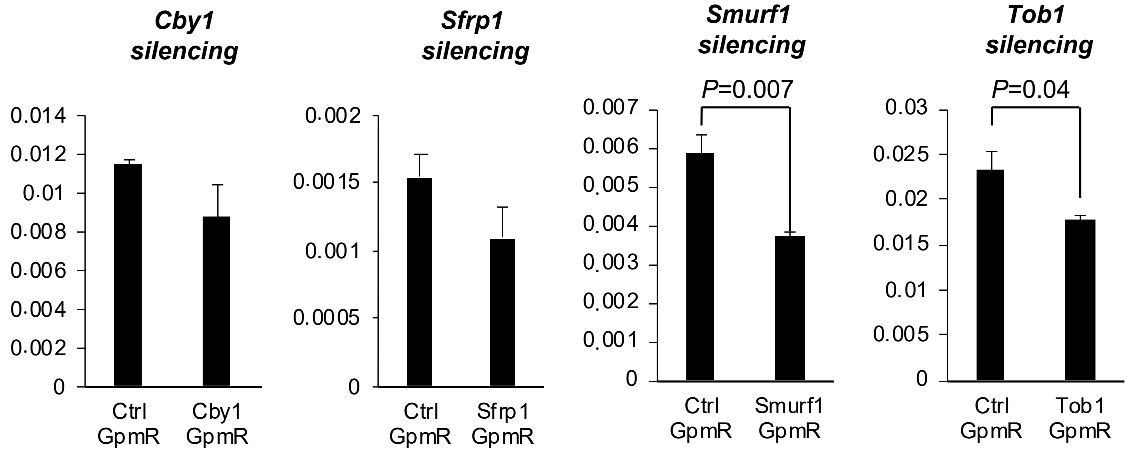

Figure S1. Silencing of the different targeted genes in C3H10T1/2 MSCs using specific GapmeRs 11 days after transfection. Ctrl stands for Control GapmeR. All values reflect averages of three different experiments independently performed. Bars represent standard error of the mean values.

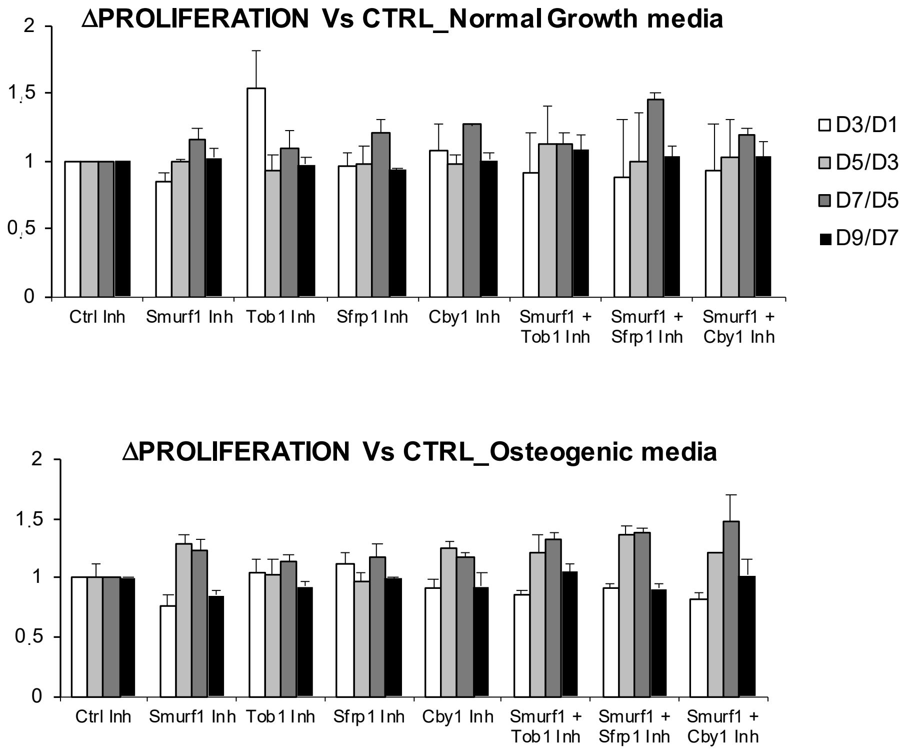

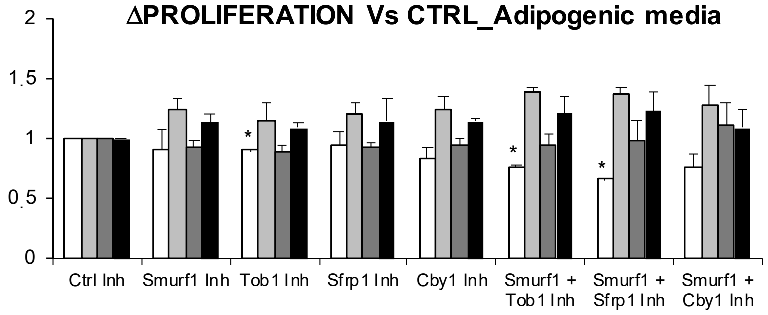

Figure S2. MTT analysis of proliferative capacity after transfection with the GapmeRs silencing different genes and the correspondent combinations. No significant differences were found in any of the culture media tested. (*n* = 3) * p ≤0.05.

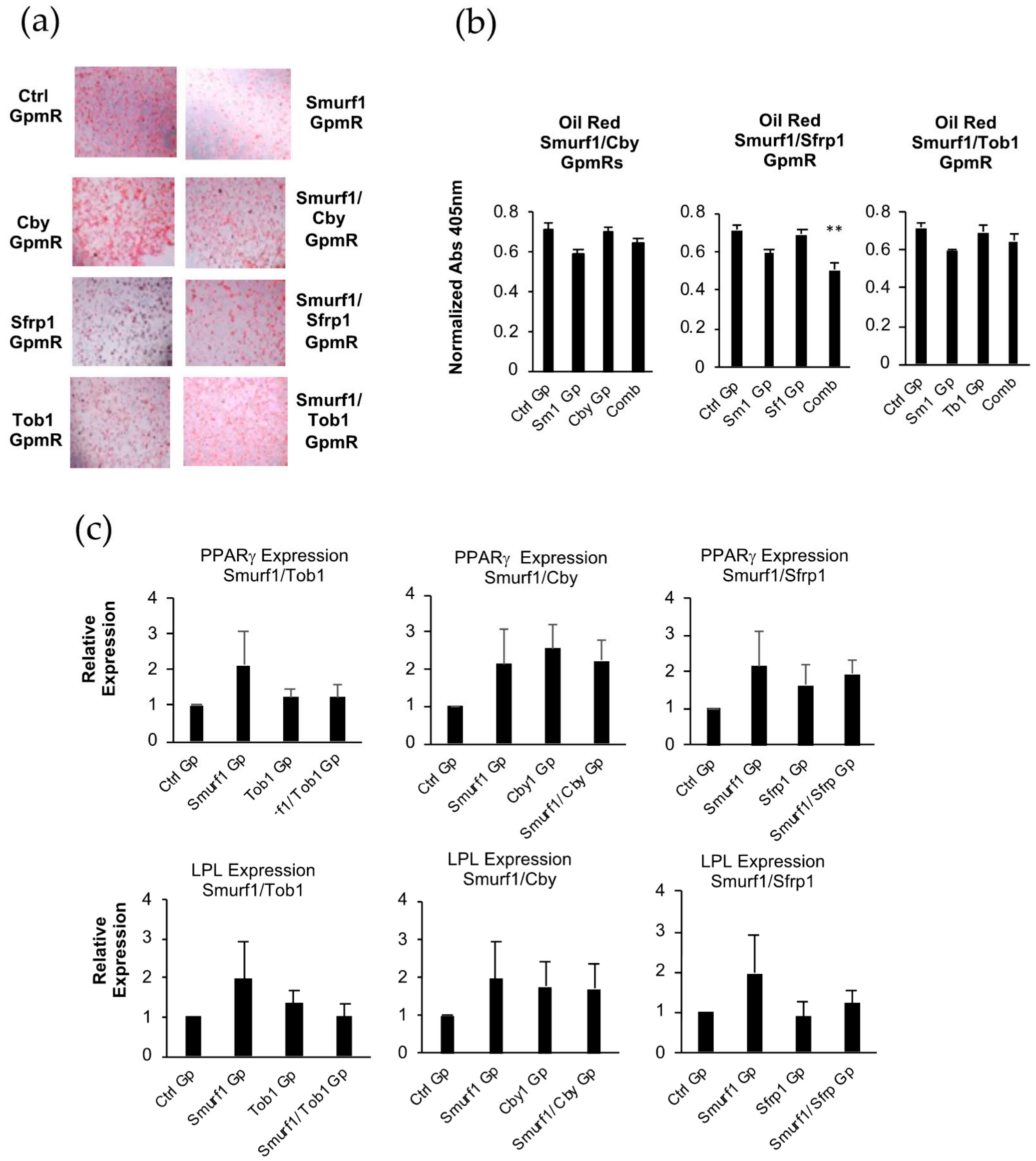

Figure S3. Adipogenic differentiation capacity of MSCs after the silencing of specific osteogenic inhibitors and their combinations. (a) Oil Red staining of cells transfected with single GapmeRs or GapmeRs combinations growing in Adipogenic media (Day 7). (a) Graphs show quantification of the staining at 550 nm. (** P≤0.005). (a) Expression of adipogenic markers *Pparγ* and Lipoprotein lipase (*Lpl*) in cells transfected with the different GapmeRs and GapmeR combinations at day 7 of adipogenic differentiation (n=3). (**P≤0.005).

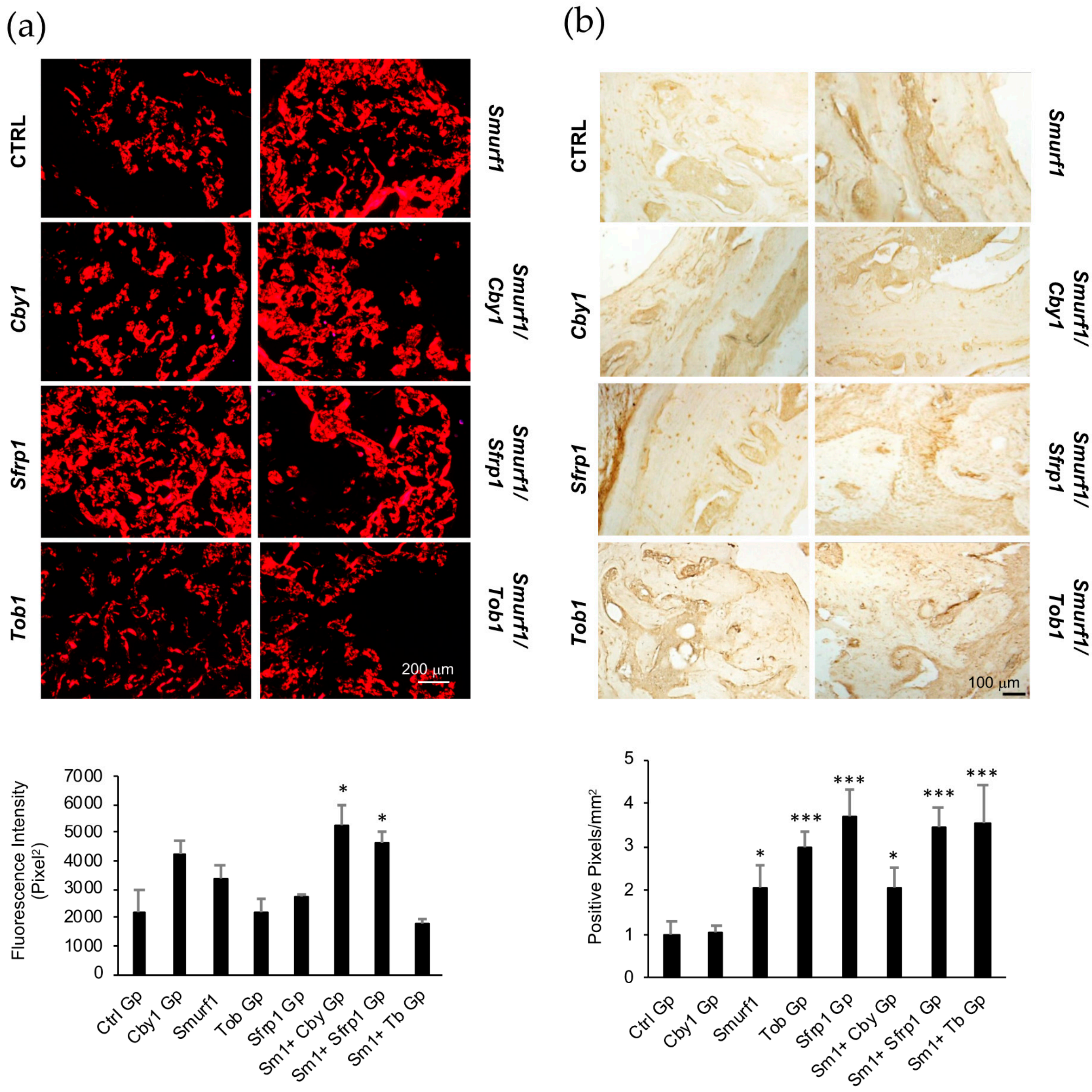

Figure S4. ***In vivo*** **analysis of the pro-osteogenic effect of the silencing of key osteogenic inhibitors in MSCs** (a) Tissue sections of each of the scaffolds analyzed stained with Sirius Red and observed under polarized light (b) Osteocalcin (OCN) immunohistochemistry. Graph shows quantification of signal intensity in the different histological samples performed using ImageJ. One implant of each type was quantified. Values correspond to average of at least six different areas measured in each of the implants. Error bars represent standard error of the mean values. (*$P \leq 0.05$; ***$P \leq 0.0005$).
